# Venetoclax plus hypomethylating agents versus intensive chemotherapy for hematological relapse of myeloid malignancies after allo-HSCT

**DOI:** 10.3389/fonc.2023.1137175

**Published:** 2023-03-23

**Authors:** Zhangjie Chen, Sisi Zhen, Tingting Zhang, Yuyan Shen, Aiming Pang, Donglin Yang, Rongli Zhang, Qiaoling Ma, Yi He, Jialin Wei, Weihua Zhai, Xin Chen, Erlie Jiang, Mingzhe Han, Sizhou Feng

**Affiliations:** ^1^ State Key Laboratory of Experimental Hematology, National Clinical Research Center for Blood Diseases, Haihe Laboratory of Cell Ecosystem, Institute of Hematology & Blood Diseases Hospital, Chinese Academy of Medical Sciences & Peking Union Medical College, Tianjin, China; ^2^ Tianjin Institutes of Health Science, Tianjin, China

**Keywords:** venetoclax, myeloid malignancy, acute myeloid leukemia, allo-HSCT, relapse

## Abstract

**Introduction:**

Since allogeneic stem cell transplantation (allo-HSCT) is considered one of the curative treatments for acute myeloid leukemia (AML) and myelodysplastic syndrome (MDS), hematological relapse following allo-HSCT remained a crucial concern for patients’ survival.

**Methods:**

We retrospectively compared patients who received venetoclax plus hypomethylating agents (VEN+HMA, n=23) or intensive chemotherapy (IC, n=42) for hematological relapse of myeloid malignancies after allo-HSCT. HMA selection included decitabine (n=2) and azacitidine (n=21), and combined donor lymphocyte infusion was administered to 21 and 42 patients in VEN+HMA and IC groups, respectively.

**Results:**

Median age of all patients was 39 (16-64) years old. Overall response rates, including complete response (CR), CR with incomplete recovery of normal neutrophil or platelet counts (CRi) and partial response (PR), were not significantly different between VEN+HMA and IC groups (60.1% versus 64.3%, P=0.785). CR/CRi rate was 52.2% in VEN+HMA and 59.5% in IC group (P=0.567). The rate of relapse after response was 66.7% in VEN+HMA group and 40.7% in IC group (P=0.176). Median overall survival was 209.0 (95%CI 130.9-287.1) days for VEN+HMA group versus 211.0 (95%CI 28.7-393.3) days for IC group (P=0.491). The incidence of lung infection (17.4% versus 50.0%, P=0.010), thrombocytopenia (73.9% versus 95.2%, P=0.035) and acute graft-versus-host disease (aGvHD) (50.0% versus 13.0%, P=0.003) was significantly higher in IC group.

**Discussion:**

In conclusion, VEN+HMA is not inferior to IC regimen in terms of improving response and survival, and is associated with a lower incidence of adverse events and aGvHD. However, further research is required to enhance long-term survival.

## Introduction

As a curative therapies, allogeneic stem cell transplantation (allo-HSCT) plays a crucial role in treating acute myeloid leukemia (AML) and high high-risk myelodysplastic syndrome (MDS), particularly for prolonging relapse-free survival and overall survival in patients with intermediate- and poor-risk AML (1). However, up to half of the patients may experience post-transplantation relapse, depending on disease status and patients’ characteristics (2, 3). Relapse often occurs during the 3-6 months following transplantation, with an overall survival of only 19% at 2 years (4). Intensive chemotherapy, donor lymphocyte infusion and second-HSCT have been utilized without significant success (5–8), indicating a need for further investigation of appropriate treatment protocols for relapse of myeloid malignancies after allo-HSCT.

BCL-2 and its inhibitors have been the subject of increasingly deepened hematological research, starting with the study of follicular lymphoma conducted by Fukuhara et al. (9) Venetoclax, the most clinically promising BCL-2 inhibitor, has been granted approval by FDA in combination with hypomethylating agents (HMA) for the treatment of newly diagnosed AML in patients not tolerant to intensive chemotherapy. Additionally, recent studies have demonstrated the impressive efficacy of venetoclax plus intensive chemotherapy for newly diagnosed and relapsed/refractory (R/R) AML (10, 11). The combination treatment of venetoclax and HMA in R/R AML patients has also been reported with varying remission rates and survival (12–15). However, its safety and effectiveness compared to other regimens in post-transplantation relapse has yet to be determined. In this retrospective study, we investigated 65 patients treated with either venetoclax plus hypomethylating agents (VEN+HMA) (n=23) or intensive chemotherapy (IC) (n=42) for hematological relapsed myeloid malignancies after allo-HSCT and compared response, survival, graft-versus-host disease (GvHD) and adverse events between the two regimens.

## Methods

### Patients

A retrospective analysis of clinical data was performed on 65 patients diagnosed with relapse of myeloid malignancy after allo-HSCT, who were treated at the Institute of Hematology and Blood Diseases Hospital, Chinese Academy of Medical Sciences and Peking Union Medical College, between November 2013 and December 2022. The study included 23 patients who received VEN+HMA and 42 patients who received IC. Patients who were initially diagnosed with primary or secondary AML or MDS and experienced hematological relapse after allo-HSCT were included in the study, while patients with severe organic dysfunction were excluded. Risk stratification, diagnosis of relapse and response criteria were according to European Leukemia Network 2017 criteria (16). Overall response rate (ORR) was defined as CR+CRi+PR. MRD positivity is defined as >0.01% myeloid blasts detected by multiparameter flow cytometry or >0.001% leukemia-associated genes detected by RT-qPCR. This study was approved by the ethical committee of the Institute of Hematology and Blood Diseases Hospital, Chinese Academy of Medical Sciences and Peking Union Medical College, and informed consent forms were obtained from all patients.

### Treatments and efficacy evaluation

Azacitidine (50mg/m^2^/d for 5 days) was used as prophylactic therapy in 4 patients after allo-HSCT. All relapsed patients discontinued immunosuppressants after diagnosis. In VEN+HMA group, venetoclax was gradually increased to a maximal dose of 400mg/d in 3 days and each treatment cycle was 14-28 days. Combined hypomethylating agents include azacitidine (75mg/m^2^/d for 7 days) or decitabine (20mg/m^2^/d for 5 days). Furthermore, eleven patients in VEN+HMA group received low-dose cytarabine (20 mg/m^2^ twice daily) for 14 days. Patients in IC group received CLAG or FLAG (cladribine 5mg/m^2^/day or fludarabine 30mg/m^2^ plus cytarabine 1-2g/m^2^/day plus G-CSF 5 ug/kg for 5 days) or IDAC, including cytarabine 1 g/m^2^/q12h plus mitoxantrone 8-10 mg/m^2^/d or idarubicin 8-12 mg/m^2^/d or daunorubicin 45-60 mg/m^2^/d or amsacrine 100 mg/m^2^/d for 3 days. Previous unsuccessful regimens were avoided in the selection of IC regimens. Donor lymphocyte infusion (DLI) was obtained from previously cryopreserved donor graft or donor’s peripheral blood. Concomitant DLI infusion was administered in 63 patients, and calcineurin inhibitor was administered in patients receiving DLI from haploidentical donors or matched unrelated donors (MUD) to prevent GvHD. GvHD prophylaxis was identical between the two groups. Median mononuclear cells and median CD34^+^ cells each dose were 2.13 (1.22-4.00) *10^8^/kg and 0.60(0.08-2.12)*10^6^/kg in VEN+HMA group, and were 2.76 (0.96-8.33) *10^8^/kg (P=0.144) and 0.65(0.17-4.27)*10^6^/kg (P=0.442) in IC group. Bone marrow aspiration was performed after each treatment course and then continued monthly to evaluate efficacy in patients achieving complete response (CR)/CR with incomplete recovery of normal neutrophil or platelet counts (CRi). Overall survival (OS) was recorded from initiation of venetoclax or IC to last follow-up or death. Relapse-free survival (RFS) was defined as time from CR/CRi to the date of hematologic relapse or last follow-up. And Data cutoff date was January 31^th^, 2023. Treatment-related mortality (TRM) was defined as death not directly caused by relapse.

### Adverse events and GvHD

During treatment session, blood routine examination, kidney and hepatic functions were monitored in all patients. Patients with neutropenic fever underwent blood culture for pathogenic microorganisms, chest imaging examination and antimicrobial therapy. Adverse events were evaluated according to CTCAE v5.0. Acute GVHD (aGVHD) and chronic GvHD (cGVHD) were diagnosed according to Glucksberg (17) and NIH (18) criteria, respectively.

### Statistical analysis

The statistical analysis was performed using IBM SPSS (v.26) and R programming language (v 4.21). Quantitative variables were expressed as median (range), categorical variables were presented as rate and percentage. Mann-Whitney U test was performed for non-normally distributed quantitative data, Chi-square test and Fisher exact probability test were used for comparison of categorical variables. Survival analysis was conducted using Kaplan-Meier method and compared using log-rank test. Univariable and multivariable analyses were calculated *via* Cox proportional hazards regression model. Co-variables were selected using a stepwise forward procedure, and clinical factors with a P<0.1 in univariable analysis were selected to fit the multivariable model. A P<0.05 was considered statistically significant.

## Results

### Primary disease status, treatment and transplantation

Patient information is summarized in **Table 1**. No significant differences were observed between VEN+HMA and IC groups concerning age, gender, initial disease types, risk stratification, therapies before allo-HSCT, donor types and disease status at transplantation. Patients in VEN+HMA group carried FLT3-ITD (n=7), RUNX1 (n=2) and c-KIT (n=1) mutations, while those receiving IC regimen had FLT3-ITD (n=3), TP53 (n=4), ASXL1 (n=3), GATA2 (n=2) and c-KIT (n=2) mutations. Additionally, complex karyotypes were presented in 1 patient in VEN+HMA group and 6 patients in IC groups. All patients received myeloablative conditioning and GvHD prophylaxis before allo-HSCT. The majority of patients in both VEN+HMA (n=11) and IC (n=24, P=0.471) groups used matched-sibling donors (MSD). One patient in VEN+HMA group and 3 patients in IC group received azacitidine maintenance after transplantation.

**Table 1:** Baseline patients and transplantation characteristics.

Item	VEN+HMA (n=23), n (%)	IC (n=42), n (%)	P value
**Age (years), median (range)**	39 (16-60)	39.6 (16-64)	0.842
**Gender** Male Female	11 (47.8%) 12 (52.2%)	22 (52.4%) 20 (47.6%)	0.725
**Initial disease** Primary AML Secondary AML MDS MDS-MLD MDS-EB-2	19 (82.6%) 1 (4.3%) 3 (13.0%) 3 (13.0%) 0 (0)	28 (66.7%) 7 (16.7%) 7 (16.7%) 0 (0) 7 (16.7%)	0.289
**ECOG score** 0 1 2 NA Median (range)	12 (55.0%) 5 (21.7%) 3 (13.0%) 2 (8.7%) 0 (0-2)	11 (26.2%) 14 (33.3%) 3 (7.1%) 14 (33.3%) 1 (0-2)	0.306 0.337
**ELN 2017 risk stratification** Favorable Intermediate Adverse NA	1 (4.3%) 15 (65.2%) 6 (26.1%) 1 (4.3%)	5 (11.9%) 20 (47.6%) 12 (28.6%) 5 (11.9%)	0.436
**Pre-transplant treatment** Intensive chemotherapy Decitabine exposure Azacitidine exposure Venetoclax exposure Median lines of therapies (range)	20 (87.0%) 5 (21.7%) 6 (26.1%) 3 (13.0%) 3 (0-5)	34 (81.0%) 7 (16.7%) 5 (11.9%) 0 (0) 3 (0-6)	0.786 0.865 0.266 0.075 0.615
**Time from diagnosis to transplant (days), median (range)**	167 (24-343)	170.5 (41-801)	0.661
**Disease status at transplant** CR/CRi MRD- PR NR MDS	16 (69.1%) 12 (52.2%) 0 (0) 4 (17.4%) 3 (13.0%)	28 (66.7%) 16 (38.1%) 4 (9.5%) 3 (7.1%) 7 (16.7%)	0.286 0.811 0.273
**Donor type** Haploidentical donor MSD MUD	10 (43.5%) 11 (47.8%) 2 (8.7%)	15 (35.7%) 24 (57.1%) 3 (7.1%)	0.771 0.538 0.471 0.793

VEN, venetoclax; HMA, hypomethylating agent; IC, intensive chemotherapy; AML, acute myeloid leukemia; MDS, myelodysplastic syndrome; MDS-MLD, MDS with multilineage dysplasia; MDS-EB, MDS with excess blasts; ECOG, Eastern Cooperative Oncology Group; ELN, European leukemia network; CR, complete remission; CRi, CR with incomplete hematologic recovery; MRD, minimal residual disease; PR, partial response; NR no response; MSD, matched sibling donor; MUD, matched unrelated donor.

### Relapse and treatment

Relapse and treatment information is displayed in **Table 2**. One patient in VEN+HMA group suffered from skin involvement and received radiation therapy. In IC group, orbital chloroma (n=1), invasion of skin (n=2), ribs (n=1), lymph nodes (n=1) and vertebras (n=2) were observed. Two patients were treated with radiotherapy and four patients with either skin or vertebras invasion presented with concurrent bone marrow relapse. Notably, 17.4% of relapsed patients (n=4) in VEN+HMA group suffered from concomitant GVHD or lung infection (aGvHD=1, cGVHD=1, pneumocystis pneumonia =1, mycoplasma pneumonia with decreased oxygen saturation=1), while only 9.5% patients in IC group (n=4, P=0.597) had similar diseases (aGvHD=2, cGvHD=1, pulmonary mycosis=1). In VEN+HMA group, four patients used VEN+HMA as second (n=3) or third line (n=1) treatment, two of whom received previous IC regimen without response and switched to venetoclax-based regimen. Twenty-one patients received azacitidine and 2 patients used decitabine in combination with venetoclax. In addition, 11 patients in VEN+HMA group received 14-day low-dose cytarabine. In IC group, IC was the first-line treatment in 37 patients, second-line in 4 patients and third-line in 1 patient. IDAC (n=15), FLAG (n=16) and CLAG (n=11) were used. IDAC treatment included cytarabine combined with mitoxantrone (n=7) or idarubicin (n=4) or daunorubicin (n=3) or amsacrine (n=1).

**Table 2:** Relapse and treatment information.

Item	VEN+HMA (n=23), n (%)	IC (n=42), n (%)	P value
**Relapse type, n (%)** Bone marrow only Extramedullary +/- BM relapse	22 (95.7%) 1 (4.3%)	35 (83.3%) 7 (16.7%)	0.293
**Relapse within 1 years after transplantation** **Concomitant disease at relapse** Active GvHD Lung infection	12 (52.2%) 4 (17.4%) 2 (8.7%) 2 (8.7%)	27 (64.3%) 4 (9.5%) 3 (7.1%) 1 (2.3%)	0.341 0.597 0.793 0.588
**BM blasts at relapse, median (range)**	21.0 (1.5-90.0) %	20.5 (0.5-91.5) %	0.429
**Hemogram at relapse, median (range)** Median WBC, 10^12^/L Median hemoglobin, 10^9^/L Median platelet, 10^9^/L	2.9 (0.9-49.6) 105 (49-141) 69.5 (10-192)	3.9 (1.3-97.6) 115 (61-156) 57.5 (3-205)	0.424 0.131 0.625
**Post-relapse treatment before HMA+venetoclax, n (%)** IC exposure AZA exposure DAC exposure DLI Median lines of therapies, median (range)	2 (8.7%) 2 (8.7%) 1 (4.3%) 2 (8.7%) 0 (0-2)	1 (2.3%) 1 (2.3%) 2 (4.7%) 3 (7.1%) 0 (0-2)	0.588 0.588 0.588 0.793 0.466
**Median time from relapse to venetoclax+HMA/IC (days), median (range)**	6 (0-178)	4 (0-104)	0.525
**Concomitant DLI, n (%)**	21 (91.3%)	42 (100.0%)	0.122

VEN, venetoclax; HMA, hypomethylating agent; IC, intensive chemotherapy; GvHD, graft-versus-host disease; BM, bone marrow; WBC, white blood cell; AZA, azacitidine; DAC, decitabine; DLI, donor lymphocyte infusion.

### Efficacy and survival

Treatment efficacy was shown in **Table 3**. All treatment responses were achieved in one cycle. Patients who did not respond, but were medically fit and willing to receive further therapies, were switched to a different regimen. In VEN+HMA group, twelve patients (52.2%) achieved CR/CRi (CR=2, CRi=10), with 4 patients (17.4%) reaching MRD negativity. However, eight of the 12 CR/CRi patients (66.7%) relapsed later. One of the 2 patients who failed prior IC achieved CRi, MRD+. Of the eight CR/CRi patients who continued with venetoclax-based treatment, one proceeded to second allo-HSCT and was alive until last follow-up. The other 4 responders all relapsed and were either treated successfully with FLAG (n=1) or died (n=3). Of the 11 non-responders, five switched to intensive (n=2) or low-dose chemotherapy (n=3), and allo-HSCT was performed in 1 NR patient, who later died of relapse. In the IC group, twenty-five (59.5%) patients achieved CR/CRi (CR=8, CRi=17), and 12 patients (28.6%) achieved MRD negativity. Eleven of the 25 patients (40.7%) who responded later relapsed. Eleven responders continued treatment with azacitidine (n=4), venetoclax (n=3), or DLI (n=4), and 6 of 17 non-responders were treated with azacitidine (n=1), DLI (n=4) or intensive chemotherapy (n=1). No statistical significance was observed between two groups regarding response, relapse after response, treatment-related mortality and early mortality.

**Table 3:** Clinical outcomes.

Items	VEN+HMA (n=23), n (%)	IC (n=42), n (%)	P value
**Reponse status, n (%)** ** ORR** ** CR** ** CRi** ** MRD- in CR/CRi** ** PR** ** NR**	14 (60.1%) 2 (8.7%) 10 (43.5%) 4 (17.4%) 2 (8.7%) 9 (39.1%)	27 (64.3%) 8 (19.0%) 17 (40.5%) 12 (28.6%) 2 (4.8%) 15 (35.7%)	0.785 0.455 0.814 0.317 0.927 0.785
**Time to response (days), median (range)**	39 (14-55)	32.5 (14-71)	0.334
**Relapse after response, n (%)**	8/12 (66.7%)	11/27 (40.7%)	0.176
**Duration of response (days), median (range)**	131 (27-394)	181 (39-1231)	0.520
**Mortality, n (%)** Day-30 mortality Day-60 mortality Day-90 mortality Treatment-related mortality	1 (4.3%) 3 (13.0%) 4 (17.4%) 1 (4.3%)	1 (2.4%) 6 (14.3%) 11 (26.2%) 9 (21.4%)	1.000 0.813 0.421 0.143

VEN, venetoclax; HMA, hypomethylating agent; IC, intensive chemotherapy; ORR, overall response rate (CR+CRi+PR); CR, complete remission; CRi, CR with incomplete hematologic recovery; MRD, minimal residual disease; PR, partial response; NR no response.

**Figure 1:**
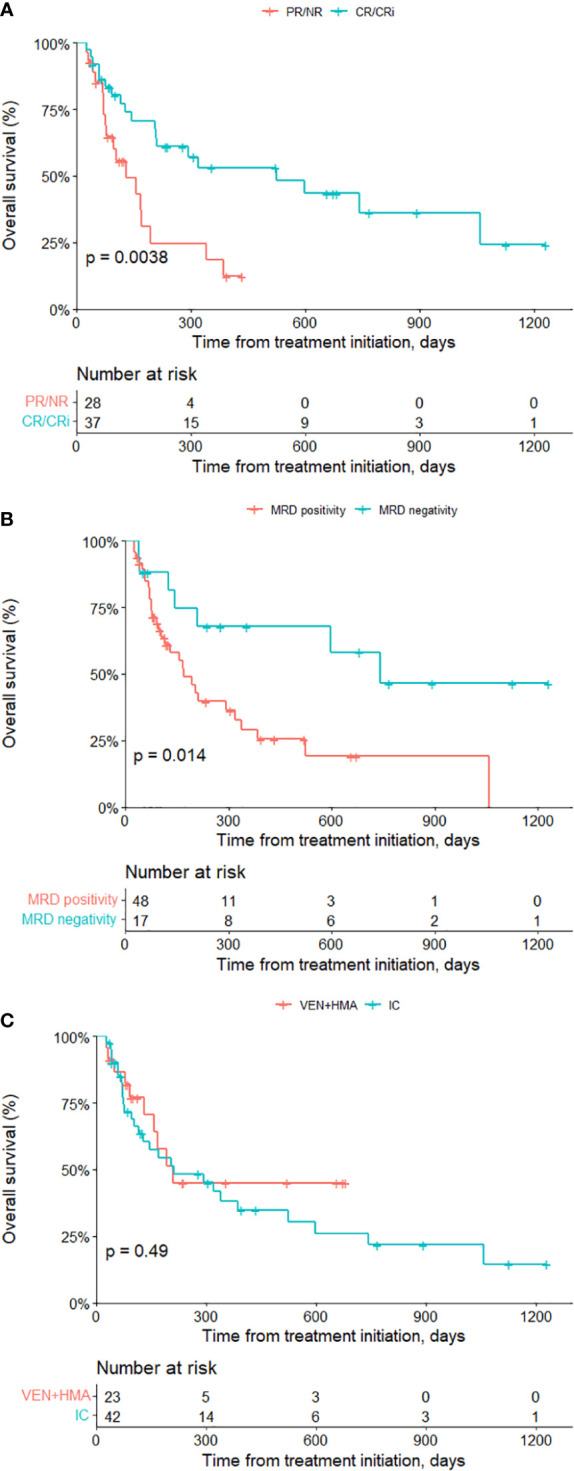
Survival analysis of all patients. Overall survival of patients achieving CR/CRi versus non-CR/CRi **(A)**, MRD negativity versus MRD positivity **(B)** and receiving venetoclax-based treatment vs. IC treatment **(C)**. VEN, venetoclax; HMA, hypomethylating agent; IC intensive chemotherapy.

Kaplan-meier survival analysis showed that achieving CR/CRi significantly improved patients’ prognosis (median OS 524 days in CR/CRi versus 130 days in PR/NR, P=0.004) (**Figure 1A**). Patients reaching MRD negativity also had significantly prolonged median OS (742 days in MRD negativity versus 169 days in MRD positivity, P=0.014) (**Figure 1B**). The median time of post-transplantation follow-up was not significant different (614 days in VEN+HMA group versus 377 days in IC group, P=0.347). Median OS was 209 days for VEN+HMA group and 211 days for IC group (P=0.491) (**Figure 1C**). In VEN+HMA group, ten patients died due to no response to regimen (n=8), relapse after CR/CRi (n=1) or severe pneumonia (n=1). In IC group, lack of response and relapse led to the death of 10 and 9 patients, respectively, and 8 patients died of infection (n=4) or GvHD (n=1) or multiorgan failure (n=3).

### Clinical factors for survival and subgroup analysis

**Figure 2:**
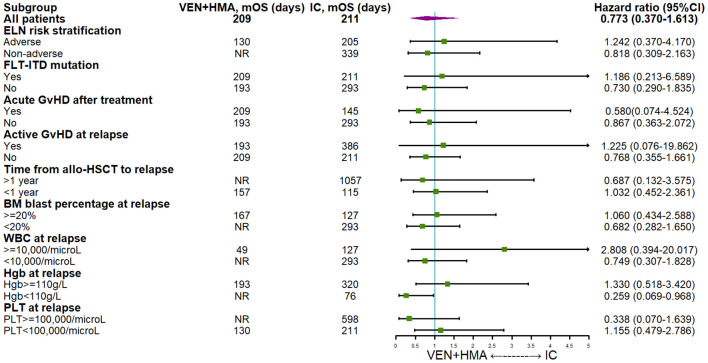
Subgroup analysis of survival VEN, venetoclax; HMA hypomethylating agent; IC, intensive chemotherapy; ELN, European leukemia network; DLI, donor lymphocyte infusion; GvHD, graft-versus-host disease; aGvHD, acute graft-versus-host disease; allo-HSCT, allogeneic hematopoietic stem cell transplantation; BM, bone marrow; WBC, white blood cell; HgB, hemoglobin; PLT, platelet.

The univariable and multivariable analysis using Cox proportional hazards regression model (**Table 4**) revealed that certain characteristics of patients’ initial diseases, including age, baseline ECOG score and adverse mutations did not significantly impact survival. TP53 mutation (HR=3.077 (95%CI 1.055-8.972), P=0.040), Grade III/IV aGvHD after treatment (HR=4.011 (95%CI 1.689-9.525), P=0.002) and time from allo-HSCT to relapse>1 year (HR=0.214 (95%CI 0.093-0.491), P<0.001) were found to have significant effects on survival in univariable analysis. Furthermore, multivariable analysis confirmed that late-onset relapse (HR=0.083 (95%CI 0.020-0.339), P=0.001) and treatment-induced grade III/IV aGvHD (HR=3.534 (95%CI 1.141-10.953), P=0.029) significantly impacted survival. In addition, multivariable analysis identified male gender (HR=4.406 (95% CI 1.599-12.140), P=0.004), FLT3-ITD mutation (HR=3.523 (95% CI 1.091-11.376), P=0.035), concomitant pulmonary infection (HR=4.060 (95% CI 1.027-16.056), P=0.046) and WBC>10,000/microL at relapse (HR=4.720 (95%CI 1.561-14.271), P=0.006) as posing significant risks. The subgroup analysis of survival was displayed in **Figure 2**, demonstrating the positive trending effect of VEN+HMA regimen in multiple subgroups, with significance observed in patients with Hgb < 110g/L at relapse.

**Table 4:** Prognostic factors for overall survival using univariable and multivariable analysis .

Items	Univariable analysis		Multivariable analysis	
	HR (95% CI)	P value	HR (95% CI)	P value
**Age** **≥40**	1.000 (0.973-1.027) 1.081 (0.566-2.064)	0.982 0.814		
**Gender (male vs. female)**	1.852 (0.951-3.607)	0.070	**4.406 (1.599-12.140)**	**0.004**
**Baseline ECOG score** **(2-3 vs. 0-1)**	2.328 (0.781-6.941)	0.129		
**Adverse ELN risk stratification** **FLT3-ITD mutation** **TP53 mutation** **ASLX1** **GATA2**	1.915 (0.943-3.890) 2.345 (0.937-5.864) **3.077 (1.055-8.972)** 1.301 (0.306-5.522) 1.584 (0.375-6.693)	0.072 0.068 **0.040** 0.722 0.531	2.469 (0.904-6.745) **3.523 (1.091-11.376)** 0.849 (0.216-3.333)	0.078 **0.035** 0.814
**VEN-based treatment vs. IC**	0.773 (0.370-1.613)	0.493		
**GvHD at any time** **Grade III/IV aGvHD after treatment**	1.057 (0.520-2.150) **4.011 (1.689-9.525)**	0.878 **0.002**	**3.534 (1.141-10.953)**	**0.029**
**Time from allo-HSCT to relapse>1 year** **GvHD at relapse** **Pulmonary infection at relapse** **BM blasts at first relapse** **BM blasts>20% at relapse**	**0.214 (0.093-0.491)** 1.415 (0.431-4.650) 3.407 (1.024-11.334) 1.003 (0.991-1.015) 1.733 (0.882-3.407)	**<0.001** 0.567 0.046 0.679 0.111	**0.083 (0.020-0.339)** **4.060 (1.027-16.056)**	**0.001** **0.046**
**WBC at relapse** **WBC>10,000/microL**	1.002 (0.985-1.020) 2.054 (0.921-4.579)	0.796 0.078	**4.720 (1.561-14.271)**	**0.006**
**Hgb at relapse** **Hgb<110g/L**	0.993 (0.980-1.005) 1.386 (0.679-2.831)	0.260 0.370		
**PLT at relapse** **PLT<100,000/microL**	0.998 (0.992-1.003) 1.450 (0.697-3.016)	0.399 0.320		
**Concomitant DLI** **Previous HMA after relapse**	1.493 (0.204-10.947) 0.873 (0.266-2.862)	0.693 0.823		

HR, hazard ratio; CI, confidence interval; ECOG, Eastern Cooperative Oncology Group; VEN, venetoclax; IC, intensive chemotherapy; GvHD, graft-versus-host disease; DLI, donor lymphocyte infusion; aGvHD, acute graft-versus-host disease; allo-HSCT, allogeneic hematopoietic stem cell transplantation; BM, bone marrow; PLT, platelet; HMA, hypomethylating agent.

### Adverse events and GvHD

Detailed information of adverse events and GvHD is shown in **Table 5**. All patients experienced grade 3-5 adverse events during their initial course of treatment. Thrombocytopenia was the most common event in both treatment groups, but the incidence was significantly higher in IC group than in VEN+HMA group (95.2% versus 73.9%, P=0.035). Pneumonia was the most common infection, with a significant higher incidence rate in IC group (50.0% versus 17.4%, P=0.010). The incidence of bacteremia was comparable between VEN+HMA group (17.4%) and the IC group (21.4%, P=0.948), and sepsis occurred in 4.3% and 4.8% patients, respectively (P=0.588). No cases of tumor lysis syndrome, patient intolerance or medication reduction were recorded, except the reduction of venetoclax to 100mg when combined with azoles. Of the 11 patients receiving further venetoclax therapy in VEN+HMA (n=8) and IC group (n=3), grade 3-5 adverse events were observed, including thrombocytopenia (n=5), neutropenia (n=3), upper respiratory infection (n=1), urinary tract infection (n=1), elevated aminotransferase (n=1).

**Table 5:** Adverse Events and GvHD.

Events	VEN+HMA (n=23), n (%)	IC (n=42), n (%)	P value
**Grade 3-5 adverse events (CTCAE v5.0)** Infection Sepsis Lung infection Upper respiratory infection Laryngitis Gum infection Oral mucositis Anal mucositis Intestine infection Abdominal infetion Skin infection Anemia Neutropenia Thrombocytopenia Elevated aminotransferase	23 (100.0%) 1 (4.3%) 4 (17.4%) 1 (4.3%) 2 (8.7%) 1 (4.3%) 1 (4.3%) 3 (13.0%) 0 (0) 0 (0) 0 (0) 11 (47.8%) 17 (73.9%) 17 (73.9%) 0 (0)	42 (100.0%) 2 (4.8%) 21 (50.0%) 4 (9.5%) 1 (2.4%) 7 (16.7%) 5 (11.9%) 7 (16.7%) 7 (16.7%) 2 (4.8%) 2 (4.8%) 27 (64.3%) 38 (90.5%) 40 (95.2%) 8 (19.0%)	1.000 0.588 **0.010** 0.793 0.588 0.293 0.577 0.978 0.098 0.536 0.536 0.198 0.158 **0.035** 0.105
**Acute GvHD after treatment** Grade III/IV aGvHD Intestine Stage 3-4 Skin Stage 3-4 Liver Stage 3-4	3 (13.0%) 1 (4.3%) 1 (4.3%) 1 (4.3%) 3 (13.0%) 3 (13.0%) 0 (0) 0 (0)	21 (50.0%) 5 (11.9%) 11 (26.2%) 6 (14.3%) 7 (16.7%) 0 (0) 19 (45.2%) 4 (9.5%)	**0.003** 0.577 0.066 0.414 0.978 0.075 **<0.001** 0.323

VEN, venetoclax; HMA, hypomethylating agent; IC, intensive chemotherapy; CTCAE, Common Terminology Criteria for Adverse Events; GvHD, graft-versus-host disease; aGvHD, acute graft-versus-host disease; cGvHD, chronic graft-versus-host disease.

After treatment of relapse, aGvHD incidence was significantly lower in VEN+HMA group (13.0% versus 50.0% in IC group, P=0.003). Grade III/IV aGvHD was observed in one patient (4.3%) in the VEN+HMA group and five patients (11.9%) in the IC group (P=0.577). Among patients with concomitant aGvHD at relapse in VEN+HMA (n=1) and IC group (n=2), one patient in each group suffered aGvHD progression. The disease severity of the 2 patients with concomitant cGvHD did not progress during treatment.

## Discussion

Allo-HSCT is considered as one of the curative treatments for high-risk AML and MDS. Despite this, relapse after transplantation remains a significant challenge. Currently available treatments, including intensive chemotherapy, DLI, etc., were partially hindered by poor efficacy and toxicity (2, 4, 19). Previous researches on IC treatment for post-transplantation AML relapse have demonstrated CR rates from 13% to 71% and 1-year OS from 25% to 34.4% (20). A recent study including 175 patients showed a remission rate of 36% and median OS of 188 days, while early mortality within 28 days occurred in 12% patients (21). The promising efficacy of venetoclax-based treatment in newly-diagnosed AML also promoted its use in R/R AML and post-transplantation relapse of myeloid malignancies. A retrospective study analyzed the efficacy of venetoclax-combined and IC regimens in R/R AML, clinical outcomes of VEN and IC groups were 59.3% and 44.4% for ORR rate (P=0.081) and 8.9 months and 12.4 months for median OS (P=0.724), revealing the comparable remission and survival provided by venetoclax (22). In contrast, two other researches showed venetoclax-based regimen can achieve significantly improved response and OS in R/R AML compared to IC treatment (23, 24). Venetoclax combination therapy for relapse of myeloid malignancies after transplantation has been reported with a CR/CRi rate ranging from 26.9% to 47.1% and a median OS from 3.4 to 9.5 months (25–28). However, these studies lack a comparison of venetoclax versus other regimens. To address this gap, we conducted this study to compare efficacy and adverse events of different salvage regimens in 65 patients with post-transplantation relapse of myeloid malignancies. Patients included received VEN+HMA (n=23) or IC treatment (n=42).

Patients’ characteristics prior to hematological relapse did not significantly differ between the two groups, CR/CRi rates were 52.2% and 59.5% for VEN+HMA and IC groups (P=0.567) and MRD negativity rates were 17.4% and 28.6%, respectively (P=0.317). However, lung infection (17.4% versus 50.0%, P=0.010), thrombocytopenia (73.9% versus 95.2%, P=0.035) and aGvHD (13.0% versus 50.0%, P=0.003) occurred significantly more frequent in IC group. Median OS was 209.0 days in VEN+HMA group versus 211.0 days in IC group (P=0.491). Although VEN+HMA achieved noninferior response and fewer adverse events, significantly improved survival was not demonstrated in OS, early mortality rate and most subgroup analyses. Patients in our study would switch to another regimen after failing the first course of venetoclax. However, previous researches have indicated the significance of multiple cycles of venetoclax treatment, as a portion of patients may reach remission after several cycles (26, 28). In addition, an increasing number of studies have emphasized the efficacy and tolerability of venetoclax maintenance therapy (29–31). Although Kaplan-Meier (median OS not reached versus 157 days, P=0.007) and univariate analysis (HR=0.184 (95%CI 0.047-0.713), P=0.014) both revealed that patients receiving continued venetoclax achieved prolonged survival versus those without maintenance therapy, the significance could be biased since patients with better physical condition were more likely to receive further treatment. Therefore, we could only speculate that the lack of continued venetoclax treatment in our study may have partially contributed to suboptimal survival.

The study found that only one patient in VEN+HMA group and 3 patients in IC group received prophylactic azacitidine maintenance, and none experienced aGvHD after relapse and treatment. The patient in VEN+HMA group suffered disease progression, whereas 3 patients in IC group all reached CRi, but 2 of them later relapsed. Univariable cox analysis did not show difference in terms of HMA prophylaxis (HR=0.772 (95% CI 0.185-3.225), P=0.723). Besides the fact results based on limited data may not accurately assess effects, previous research suggested that regular maintenance therapy could be necessary to improve survival (31, 32). Additionally, some researches (28, 33–35) revealed negative impacts of previous HMA on VEN+HMA efficacy, while other studies (14, 15) did not. In the VEN+HMA group, none of the patients with prior HMA exposure as maintenance or pre-emptive treatment achieved CR/CRi, compared to 50.0% (10/20) of those without HMA exposure. But univariable analysis did not demonstrate any significant impact of prior HMA exposure or the usage of VEN+HMA as a first-line therapy on survival.

The role of DLI and GvHD on survival also remained controversial. Previous research has produced conflicting results, with some studies indicating a positive effect of DLI and GvHD on disease remission and survival (27), while others showing no such benefits (25, 28). Our study examined the association between concomitant DLI or GvHD and patient outcomes and found no significant improvement in survival with either factor. Nevertheless, we did observe that grade III/IV aGvHD after treatment prognosticated significantly poorer survival, particularly in IC group (HR=6.547 (95% CI 2.201-19.474), P=0.001). In addition, grade III/IV aGvHD occurred with no significant difference in MSD (2/35, 5.7%) and non-MSD recipients (4/30, 13.3%, P=0.530), indicating the importance of immunosuppressants in reducing severe aGvHD in haploidentical or MUD recipients. Our study also found that relapse combined with pulmonary infection increased risks in patients treated with VEN+HMA (HR=16.598 (95%CI 2.298-119.915), P=0.005). Therefore, we recommend initiating VEN-based regimens in relapsed patients without concomitant infection. Additionally, VEN-treated patients may be more tolerant to treatment-induced GvHD than those receiving IC.

Adverse genetic abnormalities are strongly associated with R/R AML and lead to worse survival (36–38). In this study, ELN adverse stratification only showed a trend towards reducing survival (HR=2.469 (95% CI 0.904-6.745), P=0.078). Larger studies (22, 38) with more cases of R/R AML patients have shown significant impact of ELN risk stratification on survival. However, its effect has not been clearly established in patients with post-transplantation relapse. In addition, detecting new mutations at relapse and reassessing ELN risk at that time point might more accurately indicate patients’ survival. Nevertheless, due to lack of genetic testing for every patient at relapse, we were not able to demonstrate this speculation. Furthermore, multivariable analysis revealed FLT3-ITD mutation significantly influence survival, which is consistent with other research findings (22, 39). TP53 mutation also showed such significance in univariate analysis, supporting conclusion from other articles (40, 41).

In conclusion, this retrospective study demonstrated that compared to intensive chemotherapy, venetoclax plus hypomethylating agents is an effective and safe regimen for hematological relapse of myeloid malignancies after allo-HSTC. Nevertheless, prospective researches and clinical trials are necessary to verify results, and more detailed exploration is required for maintenance therapy in responders.

## Data availability statement

The original contributions presented in the study are included in the article/supplementary material. Further inquiries can be directed to the corresponding authors.

## Ethics statement

The studies involving human participants were reviewed and approved by The ethical committee of the Institute of Hematology and Blood Diseases Hospital, Chinese Academy of Medical Sciences and Peking Union Medical College. Written informed consent to participate in this study was provided by the participants’ legal guardian/next of kin.

## Author contributions

SF and XC designed the study and revised the manuscrpt. ZC analyzed the data and wrote the manuscript. SZ, TZ, YS colleted the data. AP, DY, RZ, QM, YH, JW, WZ, XC, EJ, MH, SF provided patients to study. All authors contributed to the article and approved the submitted version.
